# The Source of Firefly Chemical Defenses: Endogenous Biosynthesis of Lucibufagins from Cholesterol

**DOI:** 10.1021/acsomega.5c06679

**Published:** 2025-09-26

**Authors:** Scott R. Smedley, Kathareeya K. Medeiros, Maha Gaber, Nicole R. Howells, David A. Posner, Faizan U. Rahim, Leif D. Deyrup, Stephen T. Deyrup

**Affiliations:** † Department of Biology, Trinity College, 300 Summit Street, Hartford, Connecticut 06106, United States; ‡ Department of Chemistry and Biochemistry, 5173 Siena College, 515 Loudon Rd., Loudonville, New York 12211, United States; § Department of Biology, 8603 University of the Cumberlands, 6191 College Station Dr, Williamsburg, Kentucky 40769, United States

## Abstract

Fireflies, also known as lightning bugs, are beetles in the family Lampyridae. Several species of North American fireflies have been investigated to determine their toxic components. The primary molecules that act as chemical defenses are steroids in the bufadienolide class, called lucibufagins (LBGs). However, whether fireflies sequester LBGs from a food source or biosynthesize them de novo has remained unclear. Here, we utilize a laboratory culture of a North American firefly, *Pyractomena borealis*, to determine whether LBGs are synthesized from cholesterol. We used mass spectrometry (MS) and nuclear magnetic resonance spectroscopy (NMR) combined with a paired feeding assay to detect the incorporation of doubly ^13^C-labeled cholesterol in two LBGs produced by *P. borealis* larvae, providing direct evidence to show the production of LBGs from dietary cholesterol.

## Graphical abstract

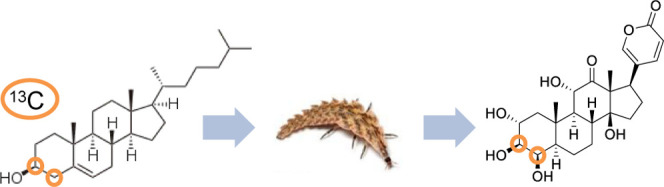

## Introduction

People have been captivated by the beauty of firefly bioluminescence for centuries;^1,2^ however, these beetles (Coleoptera: Lampyridae) have grown to be much more important to people than for aesthetics alone, including their ability to produce luciferin and luciferase, which are widely used in molecular biological and biochemical assays,^3,4^ the molecular mechanism of their luminescence,^5–7^ the evolution and role of light-production,^8–11^ their chemical defenses,^12–20^ their endosymbionts,^21,22^ and their role in ecotourism.^23–25^ Our group’s contribution to this has been focused on the chemical defenses of North American fireflies and the ecological and evolutionary implications of these defensive molecules.^20,26^

Due to their conspicuousness in all life stages and their slow movement (especially in the larval and pupal stages, which also bioluminesce), fireflies require potent means of protection from predators. In fact, evidence suggests that the light displays of these organisms originally developed as an aposematic signal (visual warning) of distastefulness to predators.^9,27,28^ In the late 1970s, pioneering research by Eisner and Meinwald revealed that North American fireflies in the genus *Photinus* contain steroidal compounds with an unusual pyrone moiety, which they dubbed lucibufagins (LBGs).^13,29,30^ Further work showed that these compounds were highly effective at deterring potential predators from birds to spiders to lizards, and that the mode of action was as a potent Na^+^/K^+^ ATPase inhibitor similar to the cardenolides ouabain and digoxin but even more toxic.^13,14,31–33^ Subsequently, it was shown that several other genera of fireflies also contain these highly active compounds including *Lucidota*,^34^ *Ellychnia*,^20^ *Lampyris*,^35^ *Pyrocoelia*,^36^ and *Photuris*,^14^ the last of which obtains them via sequestration after consuming *Photinus* using aggressive mimicry, earning them the nickname “femme fatale fireflies”. However, even with all of the ecological, evolutionary, and potential medical implications of these compounds, the origin of LBGs in fireflies has remained a mystery.^11^ Since insects are not known to produce their own cholesterol, the question has remained as to whether some fireflies can perform the biosynthesis of LBGs from dietary cholesterol, which would require conversion of the aliphatic side-chain of cholesterol to a highly oxidized pyrone ring, or whether all fireflies sequester LBGs as those in the genus *Photuris* do. Studies of the incorporation of isotopically labeled precursors into metabolites is widely considered among the most compelling evidence of endogenous biosynthesis,^37,38^ so it was decided to use this methodology. To accomplish this, we developed a method to rear the North American firefly *Pyractomena borealis* (Figure 1) in the lab. We then determined the identity of two LBGs and ran paired experiments feeding larvae either stable doubly ^13^C-isotope labeled cholesterol or unlabeled cholesterol to show whether this species can synthesize LBGs de novo from dietary cholesterol.

**Figure 1:**
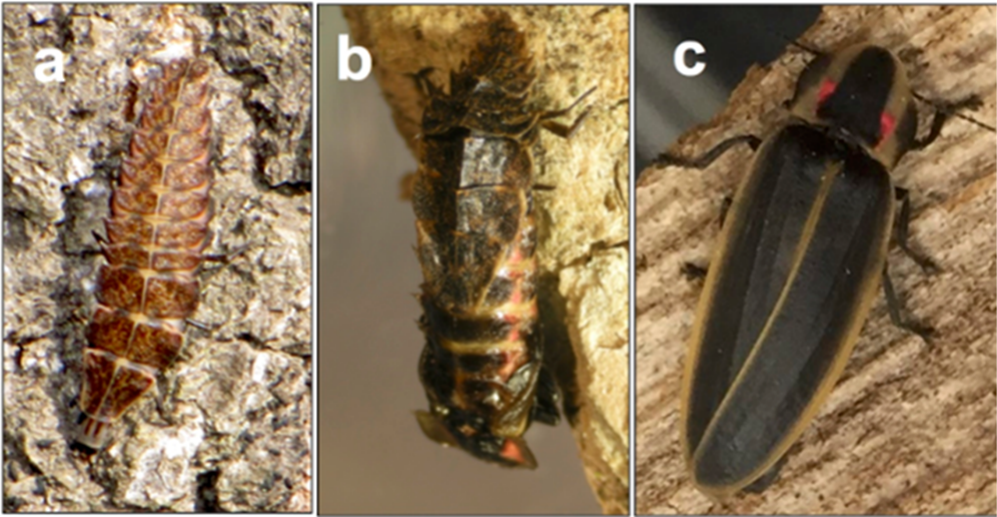
*P. borealis* at various life stages.

## Results and Discussion

Due to the close phylogenetic relationship between *P. borealis* and firefly taxa known to contain LBGs,^11,39^ it was deemed likely that they bore similar chemical defenses. Adult *P. borealis* were field collected and used to analyze the presence and identity of LBGs via NMR spectroscopy and ultra-high-performance liquid chromatography coupled with high-resolution mass spectrometry (UHPLC-HRMS). Larval *P. borealis* were field collected, paired, and mated under laboratory conditions, and their offspring were then raised for use in our LBG biosynthesis studies. To our knowledge, this is only the second record of a lab-reared culture of a North American firefly species.^40^

In order to confirm the presence of LBGs, 1D and 2D NMR spectroscopic analysis was performed on extracts of adult *P. borealis* (Figure 2). The chemical structures of LBGs vary with the producing organism, specifically oxygenation patterns on the A ring of the steroid core;^13,20,34,41^ therefore, it was important to perform NMR spectroscopic studies to identify the most abundant compounds, as mass spectrometry alone would not be sufficient.

**Figure 2:**
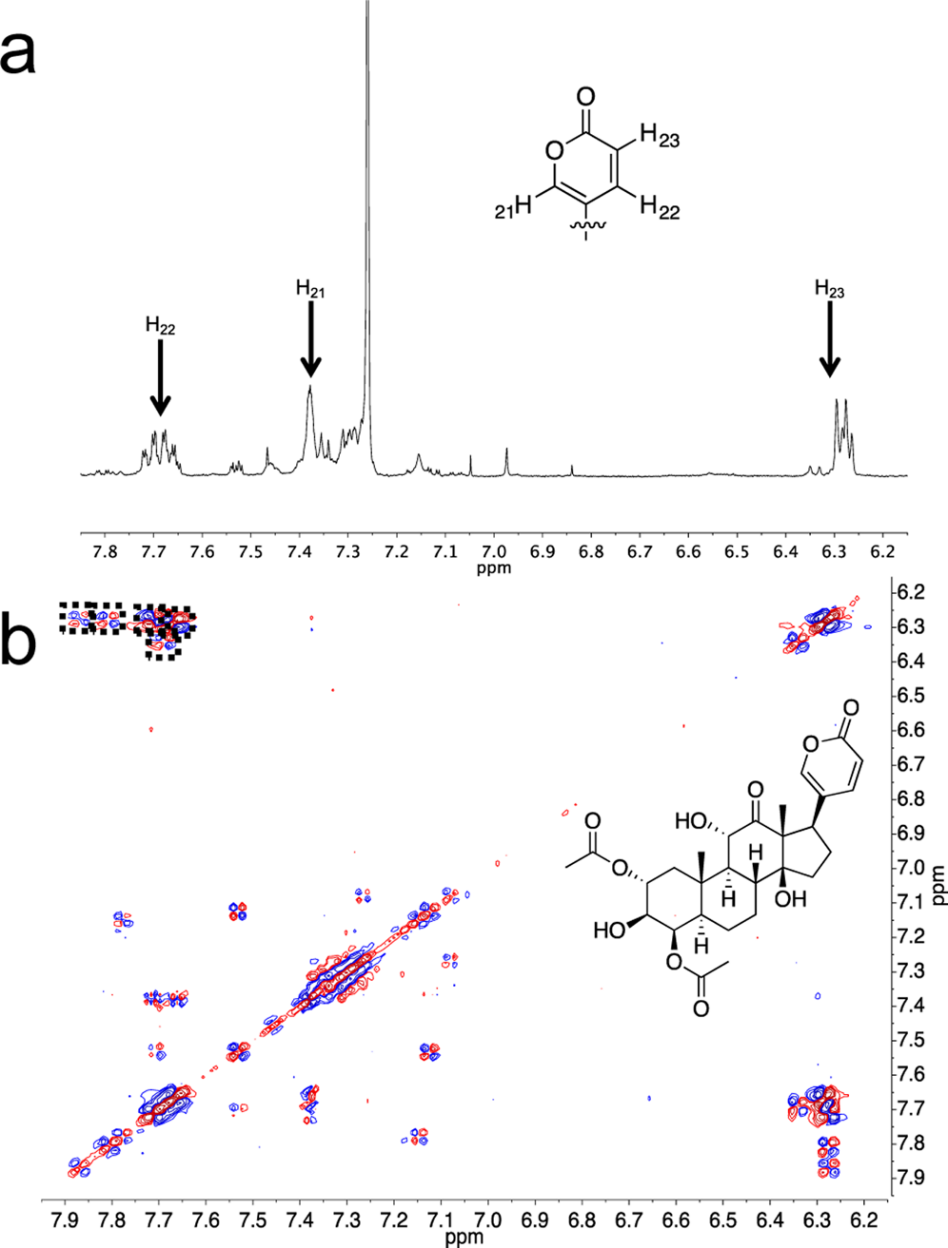
Selected portions of the ^1^H NMR and dqf-COSY, which indicated the presence of LBGs in *P. borealis*. (a) Selected portion of the ^1^H NMR spectrum of a *P. borealis* extract showing the signals from the pyrone moiety of lucibufagins. Since there are multiple lucibufagins present in the spectrum, overlapping signals are observed for each hydrogen atom in the structure. (b) Selected portion of the dqf-COSY spectrum of the *P. borealis* extract showing the presence of several overlapping signals due to pyrone moieties on different lucibufagins. Each dotted rectangle indicates a correlation from H_23_ to H_22_ on a particular lucibufagin. The structure of one of the identified lucibufagins is provided.

Analysis of the dqf-COSY spectroscopic data (Figures 2 and S2) indicated that *P. borealis* contained a mixture of at least five LBGs. This determination was made by observing five strong dqf-COSY signals for the pyrone moieties. Since pyrone rings are rare in nature and are a key characteristic of LBGs,^42^ each of these signals indicate the presence of a different LBG structure. Similar to *Photinus*, *Ellychnia*, *Lucidota*, and *Lampyris*, the major LBGs were determined to have molecular formulas of C_28_H_36_O_10_, C_28_H_36_O_10_, C_26_H_34_O_9_, and C_24_H_32_O_8_ based on analysis of UHPLC-HRMS data ([M+H]^+^ values of 533.2419, 533.2423, 491.2312, and 449.2192, respectively). Only four accurate masses for LBGs were able to be identified in the UHPLC-HRMS data due either to the possibility of coelution of isomers with the same molecular formula but different locations of the acetyl group or due to the presence of LBGs with masses that are not published in previous work on firefly chemical defenses. The structure of the major LBG (Figure 3) was determined by the analysis of 2D NMR spectroscopic data including dqf-COSY, HSQC, and HMBC (see Supporting Information for detailed structure elucidation). This LBG has a hydroxy group at the C-4 position and was identical to one isolated previously from *Lucidota atra*, providing further evidence that the genus *Pyractomena* is more closely related to the genus *Lucidota* than to *Photinus* as proposed by Martin et al.^39^ This also suggests that the major LBG in *Lampyris* probably also bears oxygenation at the C-4 position rather than the C-5 position as in *Photinus* and *Ellychnia* (Figure 4).^35^

**Figure 3:**
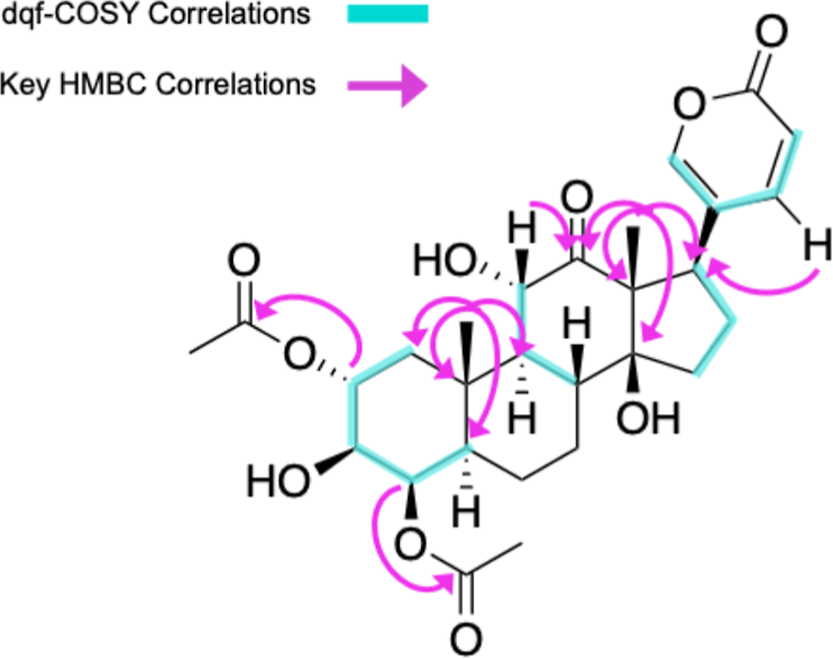
Key dqf-COSY and HMBC signals allowing for the identification of a major lucibufagin.

**Figure 4:**
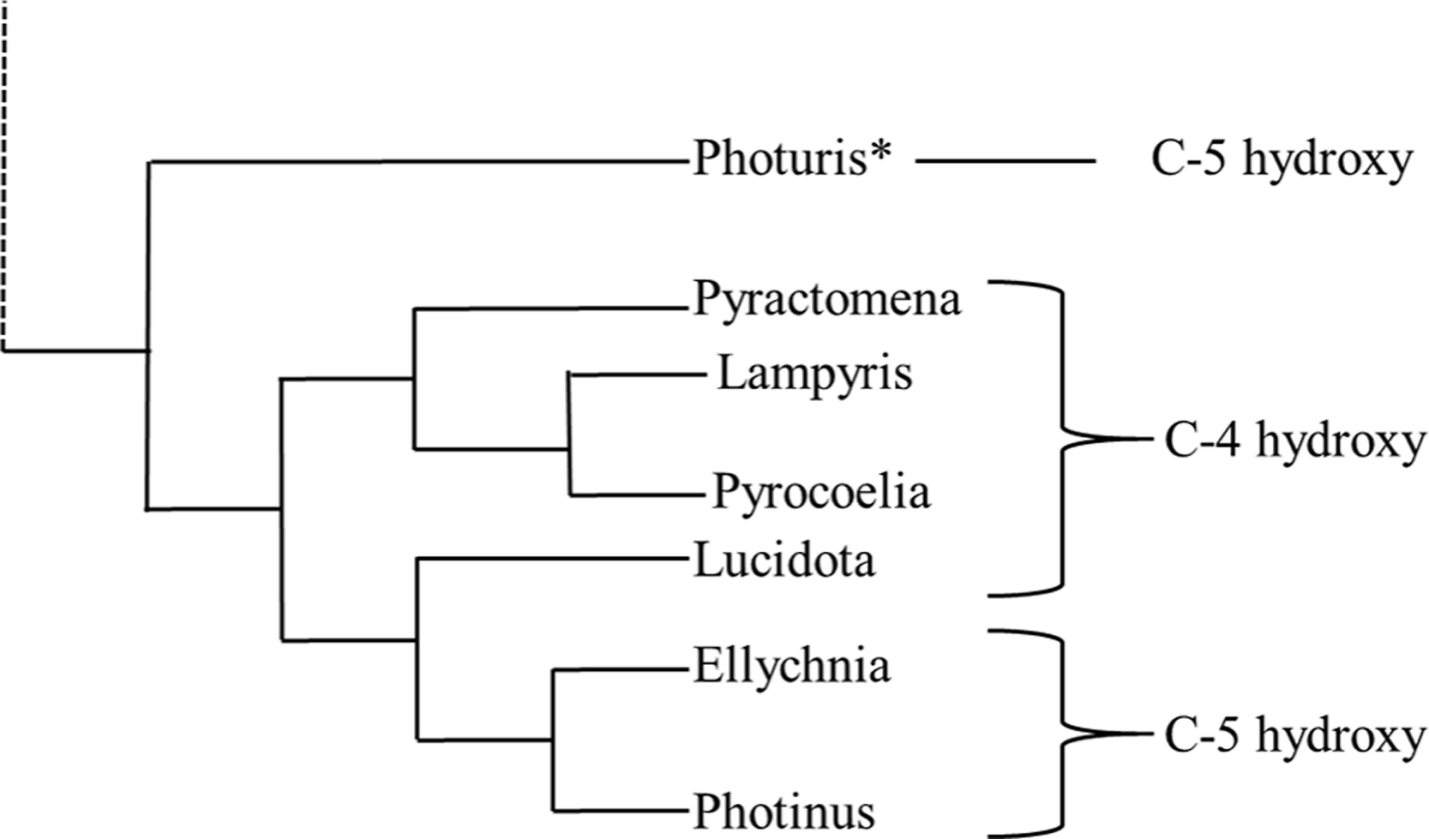
Phylogenetic relationship of fireflies shown to contain lucibufagins along with the hydroxylation pattern at the C-4 or C-5 position.

Phylogenetic relationships adapted from Martin et al.^39^ Only firefly genera that are known to possess LBGs are included. Hydroxylation at position C-4 or C-5 is noted as published in the literature, or based on our findings in this study.^13,14,20,34,36^ *Photuris* has been shown to acquire LBGs through dietary sequestration upon feeding on other firefly genera, and therefore, the ones studied chemically most likely fed primarily on *Photinus* spp. based on presence of C-5 hydroxylated LBGs.^14,26^

With the structures of the major LBGs present in *P. borealis* known, it was then possible to investigate whether these fireflies synthesize these molecules from dietary cholesterol. A study was designed where larval *P. borealis* were fed a diet supplemented with either isotopically labeled cholesterol-3,4-^13^C_2_, or nonisotopically labeled cholesterol. Mass spectrometric data were then analyzed to see whether the isotopic ratios of ^12^C to ^13^C differed between the treatment groups. Any LBGs produced using the doubly labeled cholesterol would have a mass 2 amu greater than those produced with unlabeled cholesterol since the labeled cholesterol contained enrichment of two ^13^C atoms. We observed an increase in the ratio of the [M + H]^+^+2 ion compared to the [M+H]^+^ ion in the experimental group when compared to the control group (e.g., Figure 5).

**Figure 5:**
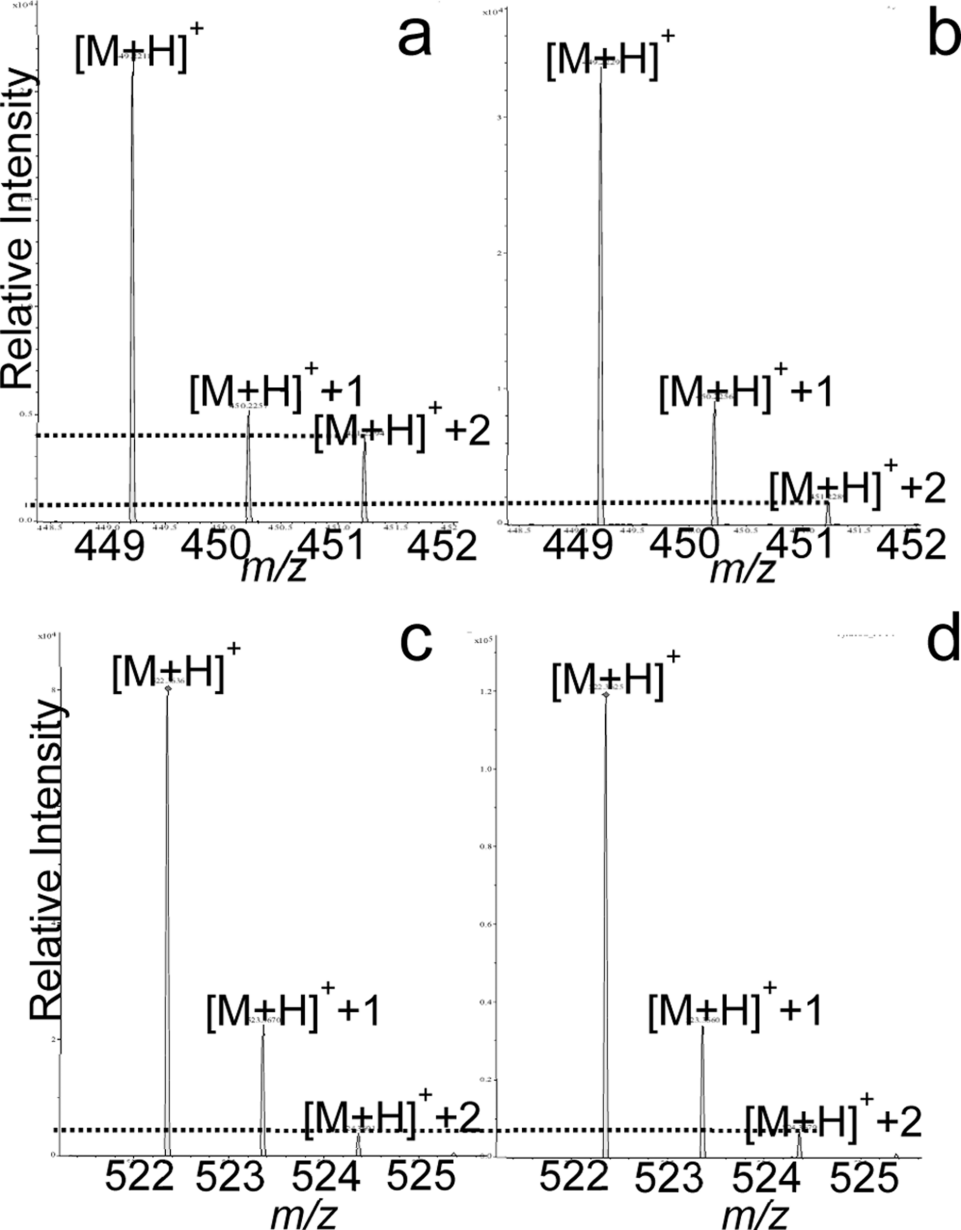
Representative results for UHPLC-MS analysis of paired *P. borealis* samples: (a) MS1 data (*t*_R_ 1.2 min) showing LBG-449 from the treatment group fed doubly ^13^C-labeled cholesterol, (b) MS1 data (*t*_R_ 1.2 min) showing LBG-449 from the treatment group fed unlabeled cholesterol, (c) MS1 data (*t*_R_ 6.7 min) showing lysophosphatidylcholine from the treatment group fed doubly ^13^C-labeled cholesterol, and (d) MS1 data (*t*_R_ 6.7 min) showing lysophosphatidylcholine from the treatment group fed unlabeled cholesterol. Dotted lines are provided for the comparison of [M+H]^+^+2 peak heights between treatments.

The data were analyzed for two different LBGs, one that contained acetate groups on the A-ring and one that did not. In both cases, the [M+H]^+^+2/[M+H]^+^ ratio was significantly increased in the group fed doubly ^13^C-labeled cholesterol. To demonstrate that the [M+H]^+^+2/[M+H]^+^ ratio was only changed for molecules biosynthesized from cholesterol, we also analyzed the MS data for 1-oleoyl-*sn*-glycero-3-phosphocholine (i.e., lysophosphatidylcholine), which showed no increase in the incorporation of ^13^C even in the group fed labeled cholesterol (Figure 6). Based on these UHPLC-HRMS data, it is clear that *P. borealis* produces LBGs from dietary cholesterol. Interestingly, it has recently been shown that *P. borealis* does not contain a large diversity of bacterial endosymbionts and that those that it does contain are unlikely to do the biochemical transformations necessary to convert cholesterol to LBGs.^22^ This indicates that the most likely option for LBG production is from cholesterol by enzymes produced by the beetles themselves, possibly cytochrome P450s.^9^

**Figure 6:**
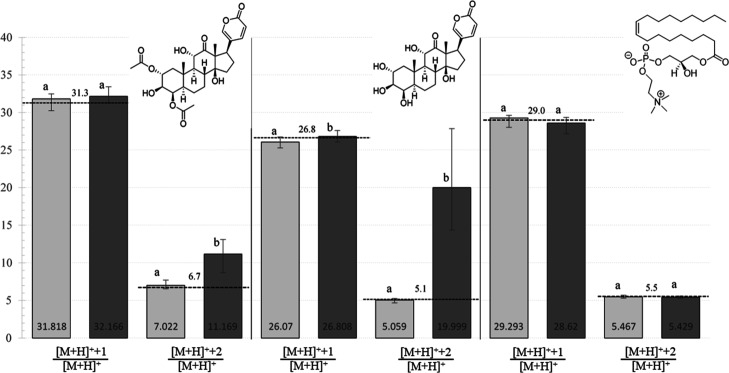
Incorporation of doubly ^13^C-labeled cholesterol into the steroid core of LBGs by *P. borealis*, as determined by UHPLC-MS analysis. *Y*-axis is the relative intensity of the [M+H]^+^+1 peak or the [M+H]^+^+2 peak (as denoted) when compared to the [M+H]^+^ peak, which was set to an intensity value of 100. Light gray bars represent values obtained from *P. borealis* larvae with unlabeled cholesterol added to their diet. Dark gray bars indicate values obtained from *P. borealis* larvae with cholesterol-3,4-^13^C_2_ added to their diet. Dashed lines with values above them indicate expected theoretical percentages of [M+H]^+^+1/[M+H]^+^ or [M+H]^+^+2/[M+H]^+^ (as denoted) based on natural ^13^C abundance. Error bars represent nonparametric 95% confidence intervals (*N* = 28, rank 8.814 to 20.186). Pairs with different letters indicate significance with a *p*-value of less than 0.05 using a Mann–Whitney *U* test.

## Conclusions

Using a culture of a North American firefly, we showed that *P. borealis* contained defensive steroids, lucibufagins (LBGs). Further we identified and characterized two LBGs present in *P. borealis* using NMR and MS techniques, which revealed that they possess compounds previously identified from *L. atra*. We also showed a correlation between the oxygenation pattern on the A-ring of LBGs and the phylogeny of fireflies that contain them. Finally, we showed that *P. borealis* produces LBGs from cholesterol obtained in their diet by feeding them doubly ^13^C-labeled cholesterol, thus confirming this widely proposed biosynthetic route to these molecules.

## Methods

### Mating of Adult *P. borealis*

Emergent adults from field-collected larvae were sexed based on light organ structure and selected for mating.^43^ A male and a female were placed in a terrarium constructed from a deep cassette (8.5 cm × 8 cm; length × depth). Apple slices were offered as a nutritional supplement. Nine mating pairs were used (mated in the lab between April 14 and May 6 2016) to obtain eggs from these parental lineages, hatched between May 30 2016 and June 13 2016.

### Rearing of *P. borealis*

Upon emergence from the eggs, larvae were kept isolated based on parental lineage. All the offspring representing a parental lineage were placed into a terrarium, which was constructed from rectangular plastic containers (20 × 14 × 10 cm; length × width × height) with mesh-screened vents in its cover as described in Archangelsky and Branham, 1998.^44^ Each terrarium’s substrate, consisting of 80% sand and 20% soil (organic layer from local field site, sifted through sieves of decreasing size (4.00 to 1.00 mm, Flinn Scientific Company, #5, #6, #7, #10 and #18)) was kept moist. Small pieces of bark and moss were added. A combination of foot muscle and viscera from crushed aquatic snails (*Physa* and *Cipangopaludina*) were offered to larvae daily. All terraria were maintained at 24 °C with a 14 h light/10 h dark cycle.

### Biosynthesis Experiment

Fifth (final) instar larvae,^44^ which range in length from 17.0 to 22.0 mm, began to pupate in late July 2016, at which time they were selected for the experiment. Each larva was transferred to its own container, a 1-ounce (29.6 mL) plastic soufflé cup (Solo Product GO6001) lined with a slightly dampened filter paper (4.5 cm diameter, P5 qualitative grade), and capped (Solo Product FK54).

A paired experimental design was employed (*n* = 28 pairs). Larvae in each pair came from the same parental lineage to control for genetic differences and potential variation in rearing conditions. Within a pair, larvae were randomly assigned to the treatment group that received a section of foot muscle from *Cipangopaludina* (1.5 mm × 5 mm, *d* × *l*) coated with labeled cholesterol-3,4-^13^C_2_ (≥98%, CLM-804-0, Cambridge Isotope Lab) or the control group that received foot muscle coated with unlabeled cholesterol (≥99%, C8667, Sigma-Aldrich). Snail muscle offerings coated with labeled or unlabeled cholesterol (200 μg/offering) were offered three times over the course of the experiment for access to a total of 600 μg of added cholesterol. Each cholesterol application was achieved by four applications of 4 μL of a 12.5 μg/μL solution with acetone (≥99.5%, A18500, Thermo Fisher Scientific) as the solvent. Whole-body larval samples were collected on day 7, which allowed 24–48 h past its last meal to potentially convert cholesterol from meals to LBG. Samples were stored at −20 °C until chemical analysis.

### Identification of Lucibufagins in*P. borealis*

All 1D and 2D NMR spectroscopic data were acquired by using a Bruker Ascend 500 NMR spectrometer with a field strength of 500 MHz for ^1^H and 125 MHz for ^13^C. NMR spectra were processed using MNova 14.3.1 software (MestreLab Research S.L.) and calibrated using the residual solvent peak (δ_1H_ = 7.260 ppm/δ_13C_ = 77.16 ppm for chloroform and δ_1H_ = 3.310 ppm/δ_13C_ = 49.00 ppm for methanol).

A sample of the whole bodies of 66*P. borealis* adults was ground in a mortar and pestle and extracted with three aliquots of 10 mL of ethyl acetate, followed by three aliquots of 10 mL of methanol. The crude extracts were then dried under air flow, resuspended in CDCl_3_, and subjected to ^1^H NMR analysis. The ^1^H NMR spectra of the crude ethyl acetate and methanol extracts from *P. borealis* showed the presence of LBGs as indicated through three signals distinct to the pyrone ring (Figure S1), which have been previously used to identify the presence of bufadienolides.^20,42^ LBG-enriched fractions were made by partitioning the crude ethyl acetate and methanol extracts between acetonitrile and hexanes and then combining the acetonitrile-soluble partitions. The acetonitrile-soluble partitions were then run over a C-18 pipet column [5 3/4″ glass Pasteur pipet (Fisher Scientific), containing 2″ C-18 silica gel, 40–60 μm, 60 Å (VWR)] using a stepwise gradient of water, 50% acetonitrile in water, acetonitrile, then 2-propanol. All fractions from the C-18 column with pyrone signals in their ^1^H NMR spectrum were combined, dried under airflow, and 1D and 2D NMR spectra were acquired in deuterated solvents. 2D NMR mixture analysis using dqf-COSY, HSQC, and HMBC allowed for further elucidation of LBG structures (Figures S2–S6) as performed previously.^45^ Molecular masses and molecular formulas for the LBGs were determined via UHPLC-HRMS experiments and aided in their identification (Table S1). Although the structural details of some of the*P. borealis*LBGs remain to be determined, the structure of two of the most abundant LBGs were determined (Figures 6 and S6).

### Detection of Cholesterol Incorporation in Lucibufagins

#### UHPLC Parameters

UHPLC was performed on an Agilent 1290 Infinity Binary LC system by using an Agilent Zorbax Eclipse Plus C-18 column (2.1 × 50 mm, 1.8 μm). Separation conditions were identical to those previously reported for the analysis of LBGs from*Ellychnia corrusca*.^20^

#### HRMS Analysis

All HRMS data were obtained using a Bruker Impact HD qTOF system, which was coupled to the Agilent UHPLC system. The mass spectrometer was used in the positive-ion mode, and conditions used were identical to those previously reported.^20^ Cholesterol-3,4-^13^C_2_ incorporation into LBGs was determined by observing shifts of relative intensities of [M+H]^+^+2 to [M+H]^+^ ratios, e.g., the intensity at *m*/*z* 451 divided by the intensity at *m*/*z* 449. The expected natural relative intensity of the [M+H]^+^+2 peak for the LBG with an [M+H]^+^ of 449 was calculated to be 5.1%, and the expected natural relative intensity of the [M+H]^+^+2 peak for the LBG with an [M+H]^+^ of 533 was calculated to be 6.7% (dashed lines in Figure 6). The resolving power of the mass spectrometer used under the conditions above was approximately 10,000, so only one peak was observed for the isobaric [M+H]^+^+2 ions. So, while the intensity of the [M+H]^+^+2 peak for any sample was not solely based on ions containing two ^13^C atoms (e.g., some might contain ^18^O instead), the increase in relative intensity of the [M+H]^+^+2 peak to the [M+H]^+^ peak should be dependent upon only an increase in ^13^C atoms.

The relative intensities of [M+H]^+^+2 to [M+H]^+^ for the compound 1-oleoyl-*sn*-glycero-3-phosphocholine (lysophosphatidylcholine) were also compared between treatment groups to provide evidence that [M+H]^+^+2 to [M+H]^+^ ratios increased only in molecules biosynthesized from cholesterol. A standard of 1-oleoyl-*sn*-glycero-3-phosphocholine was purchased (Sigma-Aldrich) to compare retention time and MS fragmentation pattern with observed data to ensure confidence in the identity of the molecule in the UHPLC-HRMS data.

## Supplementary Material



## Notes

Structure elucidation narrative, NMR data used for mixture analysis, and key UHPLC-HRMS data (PDF)

## ∥

Department of Medicine, Molecular immunity unit, MRC Laboratory of Molecular Biology, University of Cambridge. Francis Crick Avenue, CB22 0QH, Cambridge, UK

## ⊥

Lydia Becker Institute of Immunology and Inflammation. Division of Immunology, Immunity to Infection & Respiratory Medicine (DIIIRM), University of Manchester. AV Hill Building, Manchester, M13 9PT, UK.

## #

HCA Houston Healthcare, 22751 Professional Dr., Suite 120, Kingwood, TX, 77339, USA.

## ¶

This article is dedicated to the memory of Scott R. Smedley (February 22, 1963-Oct. 10, 2017).
