# Supplementary material for: The Source of Firefly Chemical Defenses: Endogenous Biosynthesis of Lucibufagins from Cholesterol
Source: ACS Omega. 2025 Sep 26;10(39):45933–9. doi: 10.1021/acsomega.5c06679 (PMC12509117; doi:10.1021/acsomega.5c06679)
Supplement: Supplementary file 1 [file ao5c06679_si_001.pdf]

Supplemental Information

The Source of Firefly Chemical Defenses: Endogenous Biosynthesis of Lucibufagins from Cholesterol

Scott R. Smedley,[1] Kathareeya K. Medeiros,[1] Maha Gaber,[2] Nicole R. Howells,[2] David A. Posner,[2†] Faizan U. Rahim,[2‡] Leif D. Deyrup[3] & Stephen T. Deyrup[2*]

[1] Department of Biology, Trinity College, 300 Summit St., Hartford, CT, 06106, USA

[2] Department of Chemistry and Biochemistry, Siena College, 515 Loudon Rd., Loudonville, NY, 12211, USA

[3] Department of Biology, University of the Cumberlands, 6191 College Station Dr., Williamsburg, KY, 40769, USA

*Corresponding author – email: sdeyrup@siena.edu

†Current addresses: Department of Medicine, Molecular immunity unit, MRC Laboratory of Molecular Biology, University of Cambridge. Francis Crick Avenue, CB22 0QH, Cambridge, UK. and Lydia Becker Institute of Immunology and Inflammation. Division of Immunology, Immunity to Infection & Respiratory Medicine (DIIIRM), University of Manchester. AV Hill Building, Manchester, M13 9PT, UK.

‡Current address: HCA Houston Healthcare, 22751 Professional Dr., Suite 120, Kingwood, TX, 77339, USA

Supplemental Information Table of Contents:

1. Structure elucidation of lucibufagins (LBGs) in *Pyractomena borealis* S3
2. Fig. S1. ^{1}H NMR spectrum of crude ethyl acetate extract of *P. borealis* in $CDCl_3$ S4
3. Fig. S2. dqf-COSY spectrum of LBG-enriched fraction of *P. borealis* extract in methanol-d_4 S5
4. Fig. S3. HSQC spectrum of LBG-enriched fraction of *P. borealis* extract in methanol-d_4 S6
5. Fig. S4. Selected portion of the HMBC spectrum of LBG enriched fraction (at lower intensity) in methanol-d_4 to show aliphatic methyl correlations S7
6. Fig. S5. Full HMBC spectrum of LBG-enriched fraction (at higher intensity) in methanol-d_4 S8
7. Fig. S6. UHPLC-HRMS Chromatograms for the combined extract of five individual adult *Pyractomena borealis* specimens S9
8. Fig. S7. UHPLC-HRMS Chromatograms for the extract of a single individual larval *Pyractomena borealis* specimen in the biosynthesis experiment S10
9. Table S1. UHPLC-HRMS data for LBGs from the combined extract of five individual adult *Pyractomena borealis* specimens S11
10. Fig. S8. Structures of the known LBGs identified from *P. borealis* S12
11. References used in SI S13

Structure elucidation of lucibufagins (LBGs) in *Pyractomena borealis*:

The structure elucidation of the major LBGs present in *P. borealis* was greatly aided by comparison to the literature data[1] and having acquired the molecular formula from the mass spectrometric data (HRMS of [M+H]+ = 533.2419, $C_{28}H_{37}O_{10}$).Three major spin-systems were determined using dqf-COSY correlations, then linked using HMBC correlations (Fig. 3 in main text). Spin system C-1–C-5 was constructed based on correlations from oxymethine H-2 (δ=5.15 ppm) to H-1α (δ=1.28 ppm), H-1β (δ=2.84 ppm), and H-3 (δ=3.67 ppm), along with correlations from oxymethine H-4 (δ=5.37 ppm) to H-3 (δ=3.67 ppm) and H-5 (δ=1.44 ppm). The C-8, C-9, C-11 spin system was identified by correlations from H-9 (δ=1.14 ppm) to H-8 (δ=1.96 ppm) and H-11 (δ=4.37 ppm) while the C-15–C-17 spin system was determined by observing correlations from H-16α/β (δ=2.04 ppm) to H-15α/β (δ=1.24 ppm) and H-17 (δ=4.07 ppm). The pyrone ring spin-system was determined based on a strong correlation between H-22 (δ=7.67 ppm) and H-23 (δ=6.28 ppm), along with a weaker long-range correlation between H-22 and H-21 (δ=7.38 ppm). HMBC correlations from H_3-19 (δ=1.33 ppm) to C-1 (δ=43.7 ppm), C-5 (δ=47.8 ppm), C-10 (δ=39.1 ppm), and C-9 (δ=54.0 ppm) connected the C-1–C-5 spin system with the C-8, C-9, C-11 spin-system, while a correlation from oxymethine H-11 (δ=4.37 ppm) to C-12 (δ=213.1 ppm) extended the connectivity through the ketone moiety. Correlations from H_3-18 (δ=0.93 ppm) to C-12 (δ=213.1 ppm), C-13 (δ=61.5 ppm), C-14 (δ=85.3 ppm), and C-17 (δ=40.7 ppm), along with a correlation from H-22 to C-17, completed the connectivity of the core structure of the identified LBG. Location of the acetate functionalities was based on the downfield shifts of the oxymethine hydrogen atoms H-2 (δ=5.15 ppm) and H-4 (δ=5.37 ppm) along with the molecular formula and comparison to literature data[1].

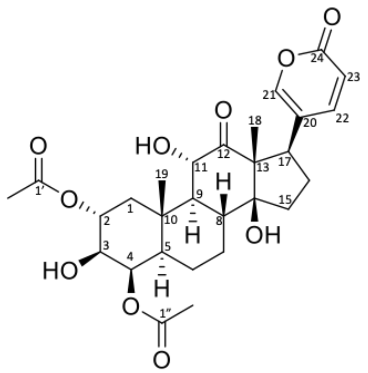

Fig. S1. ^{1}H NMR spectrum of crude ethyl acetate extract of *P. borealis* in $CDCl_3$

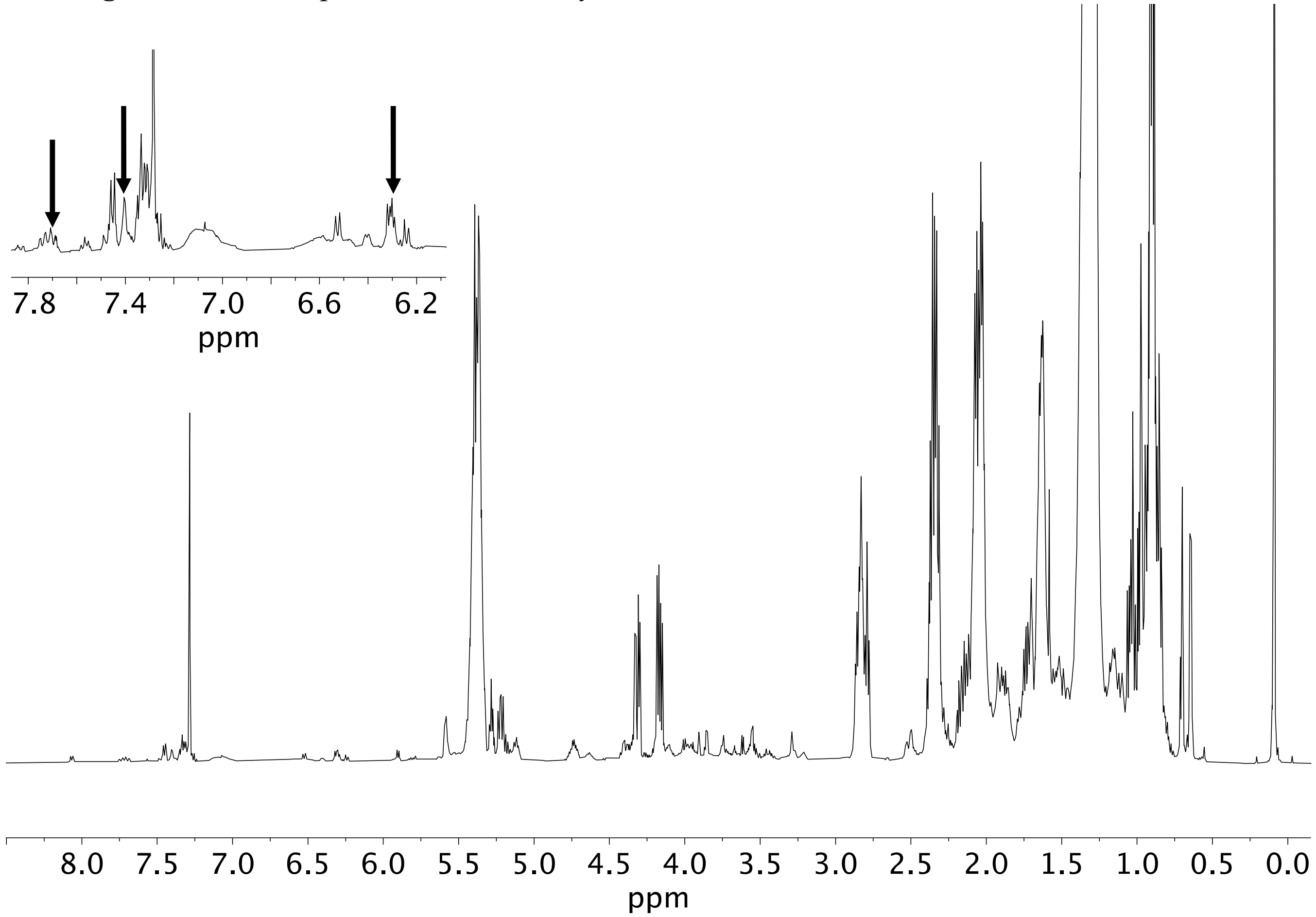

Inset is a zoomed-in portion of the region of the spectrum that contains pyrone signals (arrows).

Fig. S2. dqf-COSY spectrum of LBG-enriched fraction of *P. borealis* extract in methanol-d_4

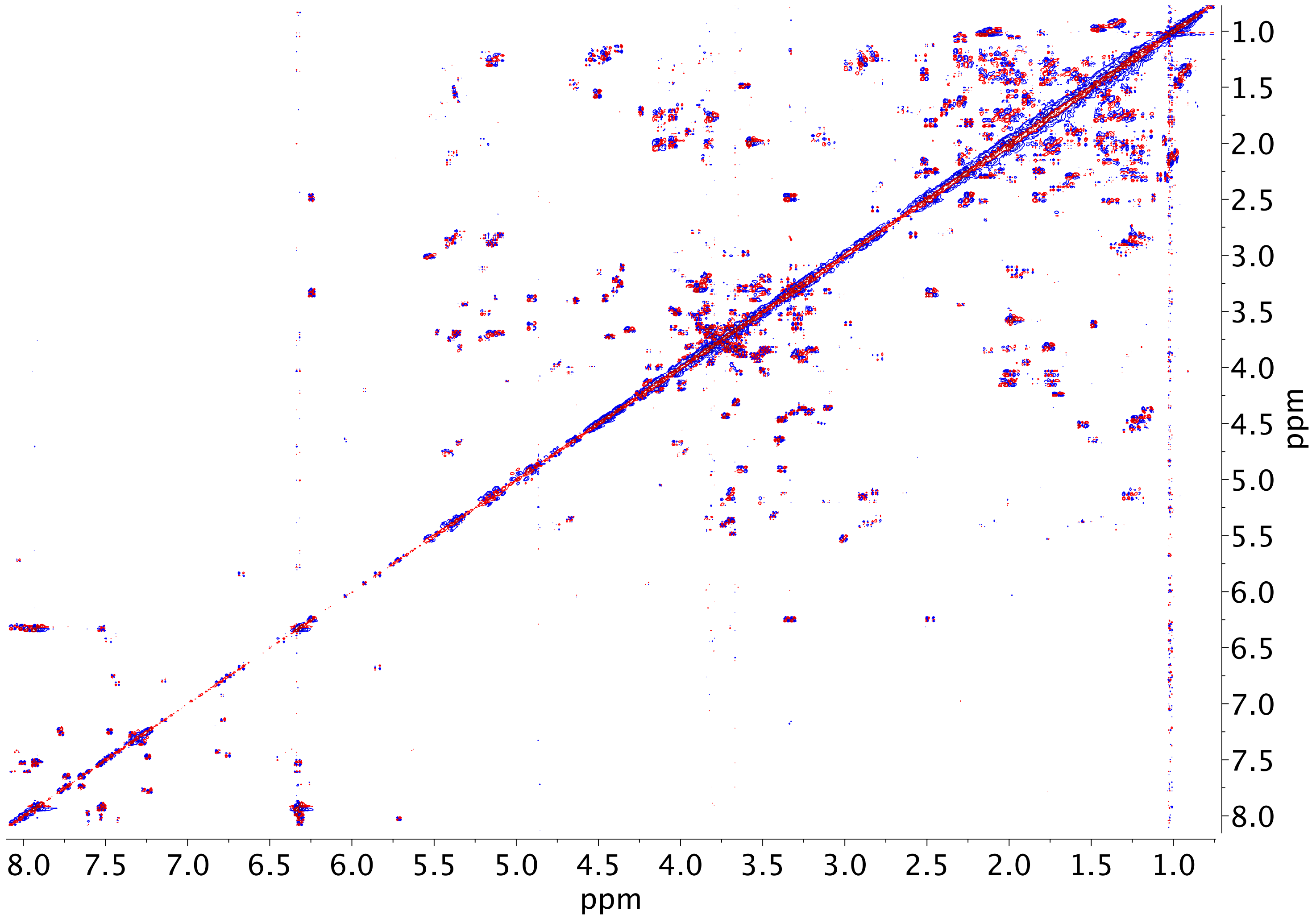

Fig. S3. HSQC spectrum of LBG-enriched fraction of *P. borealis* extract in methanol-d_4

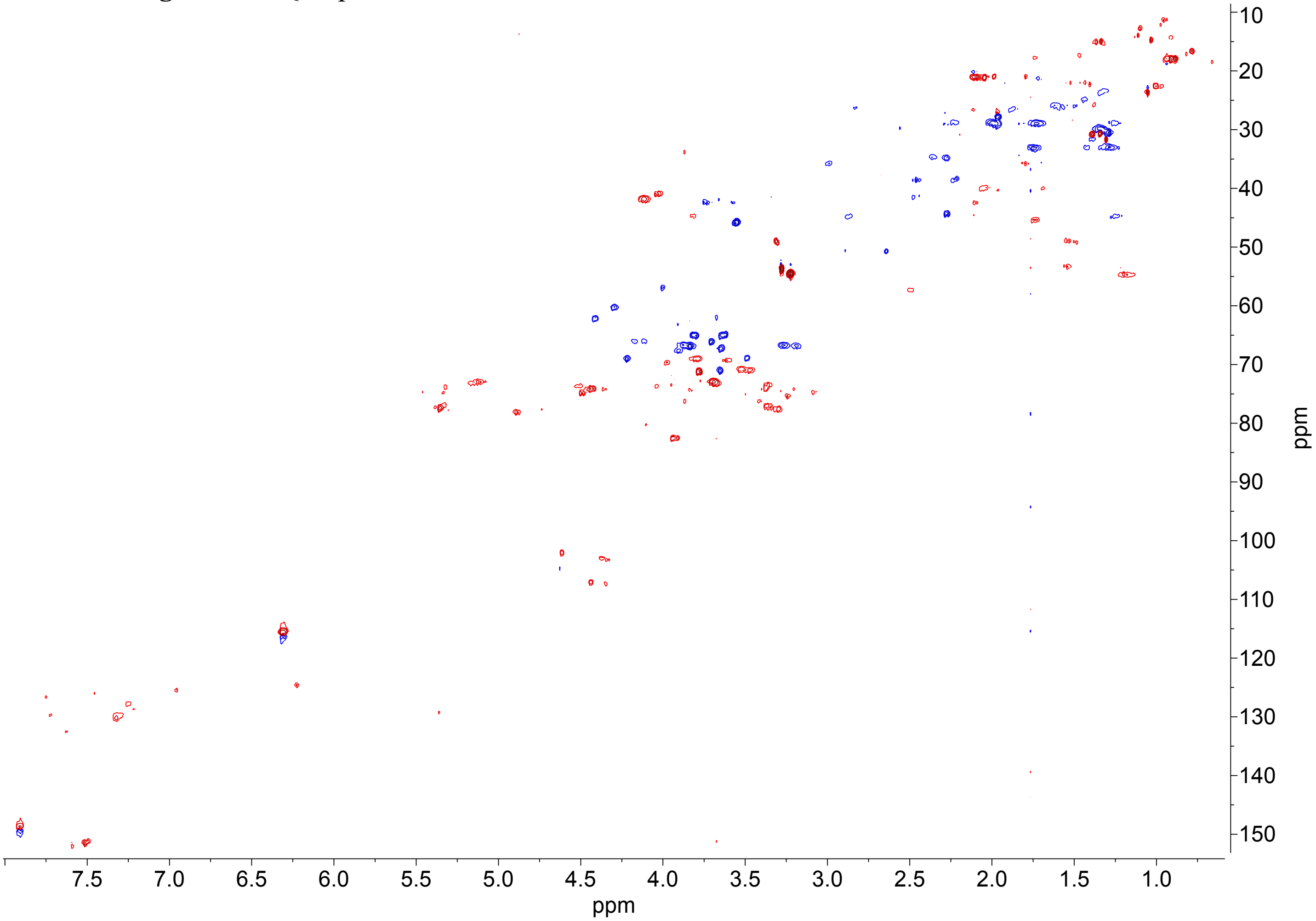

Fig. S4. Selected portion of the HMBC spectrum of LBG enriched fraction (at lower intensity) in methanol-d_4 to show aliphatic methyl correlations

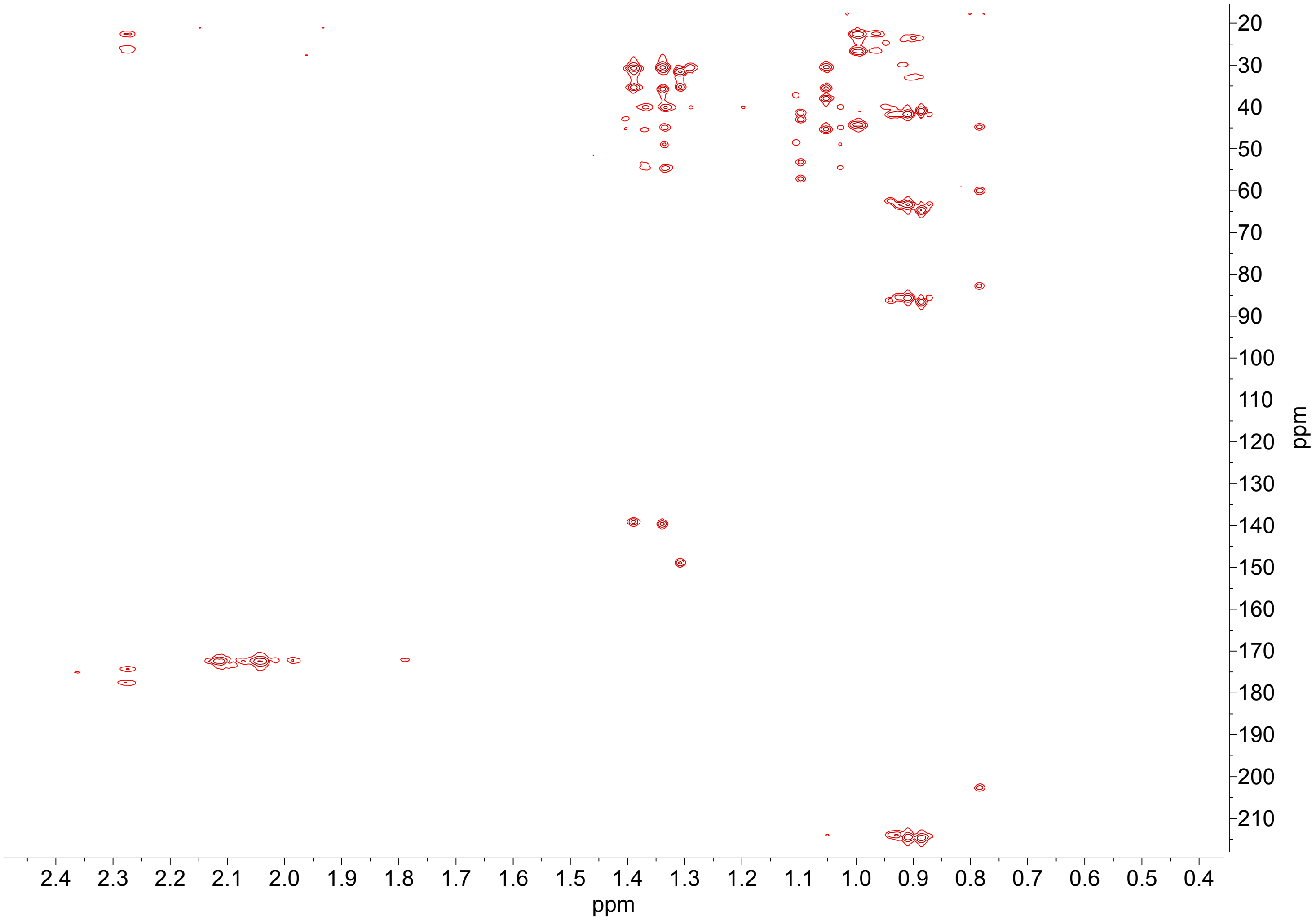

Fig. S5. Full HMBC spectrum of LBG enriched fraction (at higher intensity) in methanol-d_4

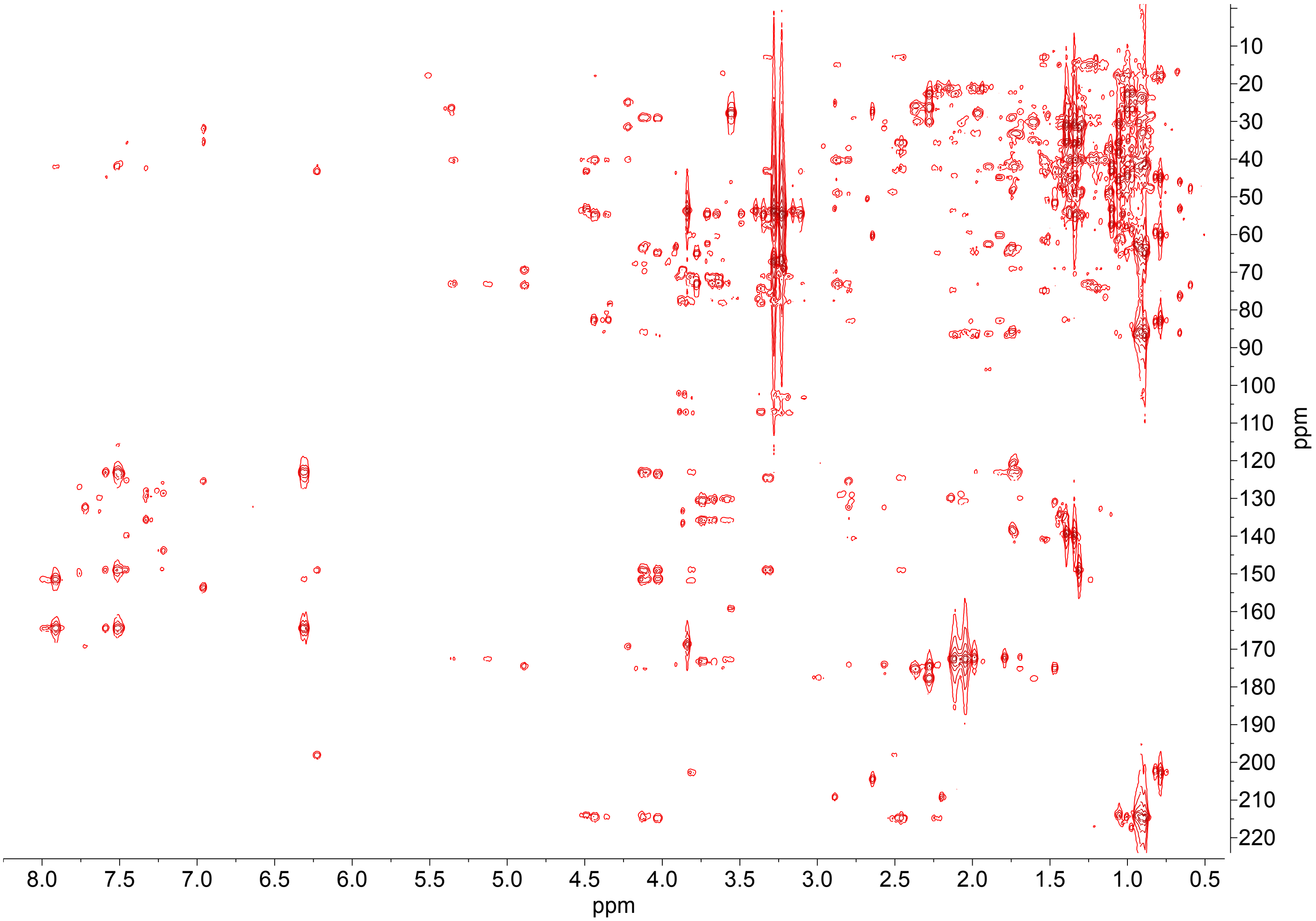

Fig. S6. UHPLC-HRMS Chromatograms for the combined extract of five individual adult *Pyractomena borealis* specimens

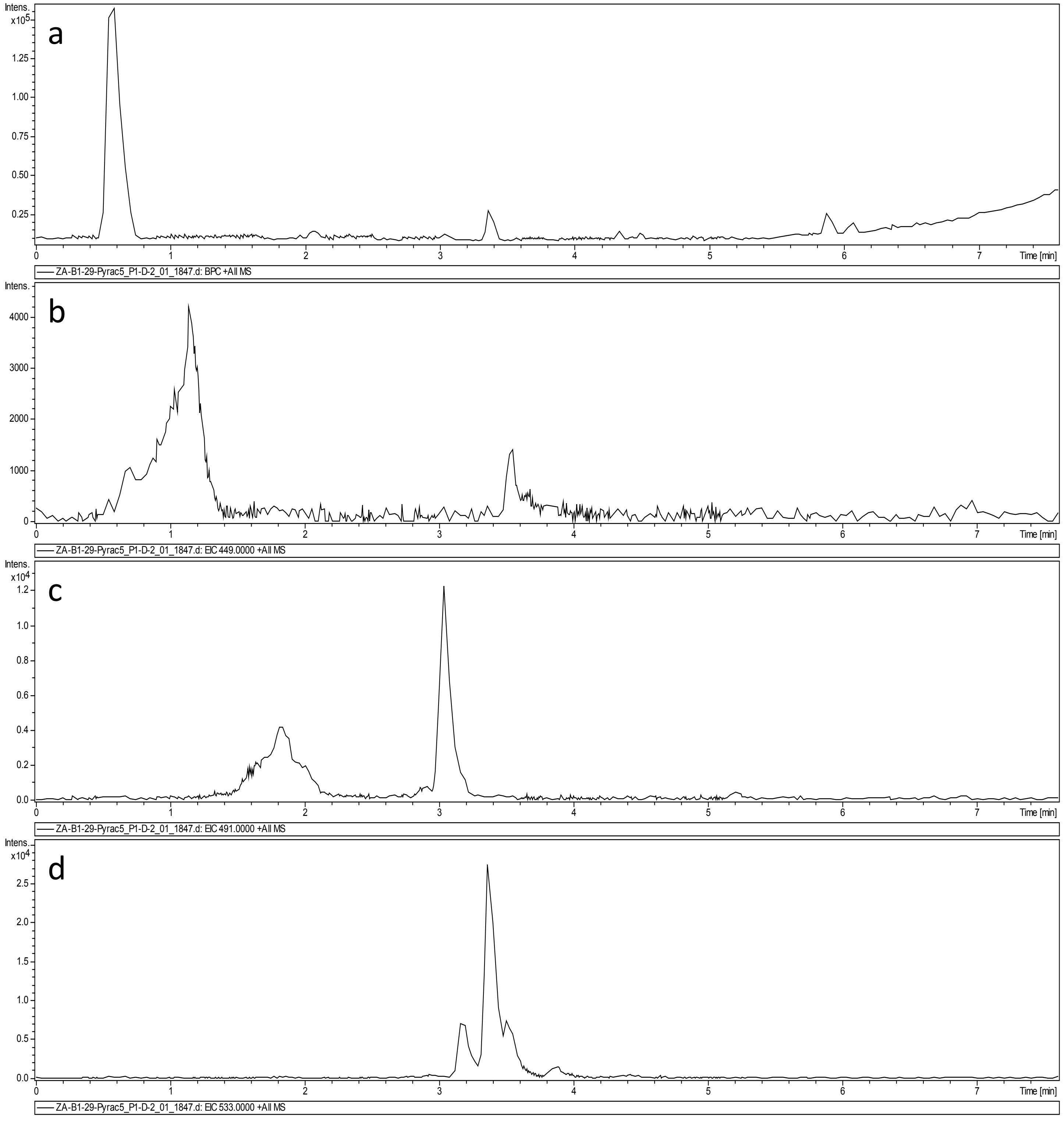

(**a**) Base peak chromatogram. (**b**) Extracted ion chromatogram for 449 (± 1 amu). (**c**) Extracted ion chromatogram for 491 (± 1 amu). (**d**) Extracted ion chromatogram for 533 (± 1 amu).

Fig. S7. UHPLC-HRMS Chromatograms for the extract of a single individual larval *Pyractomena borealis* specimen in the biosynthesis experiment

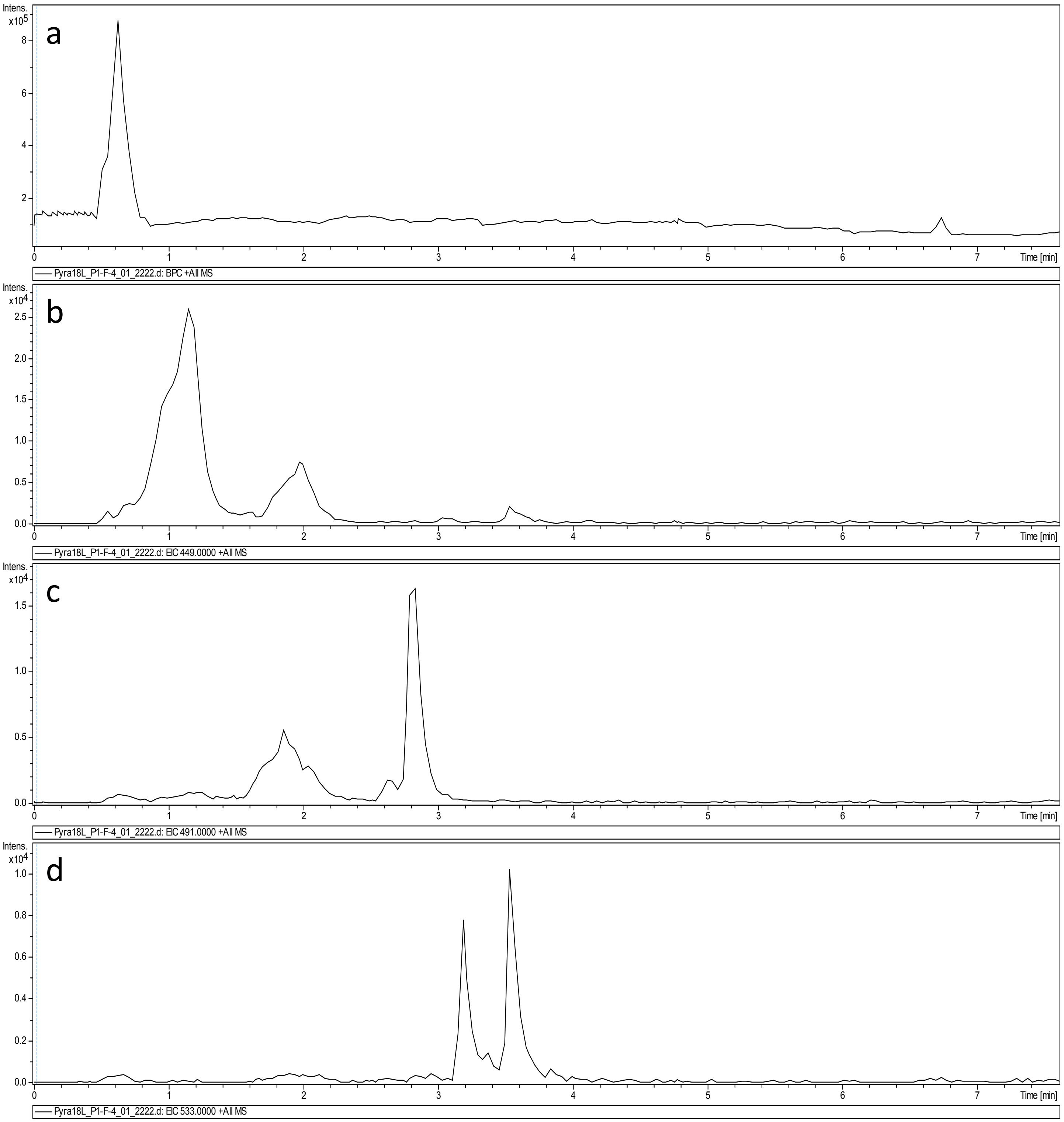

(**a**) Base peak chromatogram. (**b**) Extracted ion chromatogram for 449 (± 1 amu). (**c**) Extracted ion chromatogram for 491 (± 1 amu). (**d**) Extracted ion chromatogram for 533 (± 1 amu).

Table S1. UHPLC-HRMS data for LBGs from the combined extract of five individual adult *Pyractomena borealis* specimens

Nominal Mass	Retention Time	Accurate Mass	Formula	Calc. Mass	Error (ppm)
449	1.2 min	449.2192	$C_{24}H_{33}O_8$	449.2170	-5.0
491	3.0 min	491.2312	$C_{26}H_{35}O_9$	491.2276	-7.3
533	3.2 min	533.2419	$C_{28}H_{37}O_{10}$	533.2381	-7.0
533	3.4 min	533.2423	$C_{28}H_{37}O_{10}$	533.2381	-7.8

Fig. S8. Structures of the known LBGs identified from *P. borealis*

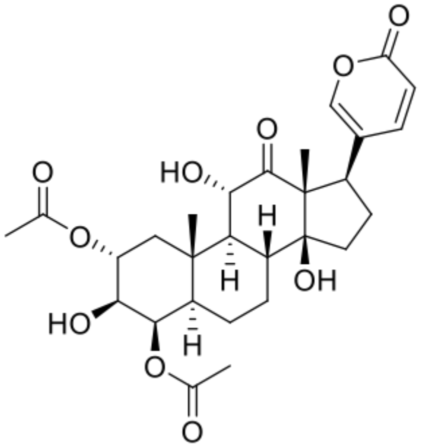

[M+H]$^+$ m/z = 533.2419

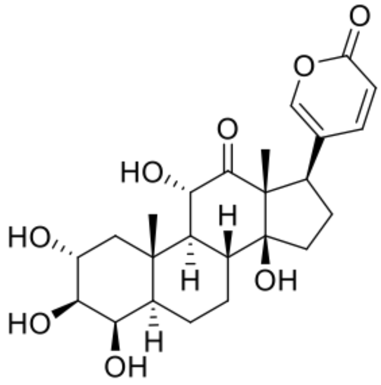

[M+H]$^+$ m/z = 449.2192

While both UHPLC-HRMS and 2D NMR spectra indicated the presence of several additional lucibufagins, these two were able to be confidently assigned based on their data and comparison to literature data.[1]

References

1. Gronquist, M., Meinwald, J., Eisner, T. & Schroeder, F. C. Exploring Uncharted Terrain in Nature's Structure Space Using Capillary NMR Spectroscopy: 13 Steroids from 50 Fireflies. *J Am Chem Soc* **127**, 10810–10811 (2005).
